# The east coast districts are the possible epicenter of severe dengue in Sabah

**DOI:** 10.1186/s40101-020-00230-0

**Published:** 2020-08-14

**Authors:** Narinderjeet Kaur, Syed Sharizman Syed Abdul Rahim, Joel Judson Jaimin, Jiloris Julian Frederick Dony, Koay Teng Khoon, Kamruddin Ahmed

**Affiliations:** 1 Department of Community and Family Medicine, Faculty of Medicine and Health Sciences, Universiti Malaysia Sabah, 88400 Kota Kinabalu, Sabah Malaysia; 2 Public Health Lab, Kota Kinabalu Public Health Laboratory, Sabah State Health Department, 88300 Kota Kinabalu, Sabah Malaysia; 3 Vector borne Unit, Sabah State Health Department, 88590 Kota Kinabalu, Sabah Malaysia; 4 Borneo Medical and Health Research Centre, Faculty of Medicine and Health Sciences, Universiti Malaysia Sabah, 88400 Kota Kinabalu, Sabah Malaysia; 5 Department of Pathobiology and Medical Diagnostics, Faculty of Medicine and Health Sciences, Universiti Malaysia Sabah, Jalan UMS, 88400 Kota Kinabalu, Sabah Malaysia

**Keywords:** Dengue, Severe dengue, Sabah, Serotype, Genotype

## Abstract

**Background:**

Malaysia recorded the highest number of dengue cases between 2014 and 2017. There are 13 states and three federal territories in Malaysia, and each area varies in their prevalence of dengue. Sabah is one of the states situated in Borneo, Malaysia. Although dengue has been increasing for the last several years, no study was being done to understand the burden and serotype distribution of the dengue virus (DENV) in Sabah. Therefore, the present study was carried out to understand the epidemiology of the dengue infection and the factors responsible for severe dengue in Sabah.

**Methods:**

Data on dengue infection were extracted from the dengue database of the state of Sabah from 2013 through 2018. DENV NS-1-positive serum samples from multiple sites throughout Sabah were sent to the state public health laboratory, Kota Kinabalu Public Health Laboratory, for serotype determination. The analysis of factors associated with severe dengue was determined from the data of 2018 only.

**Results:**

In 2013, there were 724 dengue cases; however, from 2014, dengue cases increased exponentially and resulted in 3423 cases in 2018. Increasing dengue cases also led to increased dengue mortality. The number of dengue deaths in 2013 was only five which then gradually increased, and in 2018, 29 patients died. This is an increase of 580% from 2013 to 2018. Deaths were considerably more in the districts of the east coast of Sabah compared with districts in the west coast. During the study period, all DENV serotypes could be identified as serotypes circulating in Sabah. In 2018, the predominant serotype was DENV-3. The monthly peak of dengue infection varied in different years. In the logistic regression analysis, it was identified that children were 6.5 times, patients infected with mixed serotype of DENV were 13 times, and cases from the districts of the east coast were 5.2 times more likely to develop severe dengue.

**Conclusions:**

An increasing trend of dengue infection has been observed in Sabah. The burden of dengue, severe dengue, and mortality was noted especially in the districts of the east coast of Sabah. Severe dengue was most likely developed in children, cases from the east coast, and patients infected with mixed serotype of DENV.

## Background

Dengue fever is a viral infection that is transmitted to humans by the bite of infected *Aedes* mosquitoes. Globally, every year, an estimated 390 million people are infected with dengue [1]. About 75% of these occur in Southeast Asia and Western Pacific regions [2]. Before 1970, only nine countries were endemic for dengue; however, now, 120 countries are affected indicating dengue is spreading at an alarming pace [3]. It is thought that global warming might have increased the survival and/or migration of mosquitoes into previously non-endemic areas outside the tropics. Globalization, urbanization, trade, and travels [4] might have also contributed to the spread of dengue.

Dengue infection is generally mild [5], but in 5 to 20% of cases, it may progress to severe dengue depending on the immunity of the person [4, 5]. Severe dengue is manifested by plasma leakage, hemorrhage and organ dysfunction, and even death [6]. Early supportive care can reduce mortality in severe dengue cases; however, there is no reliable method to predict progression to severe dengue. The warning signs for severe dengue have a low positive predictive value and develop only later in the disease where it might already be too late [5].

Dengue virus (DENV) is a positive-sense, single-stranded RNA virus found in the genus *Flaviviridae*. It is classified into four main serotypes (DENV-1, DENV-2, DENV-3, and DENV-4), which are distinct genetically but cause similar disease features. Infection with one of the serotypes is believed to produce durable, life-long, homotypic immunity against the particular serotype; however, it only generates partial and transient cross-protection against the other three serotypes. This allows for sequential dengue infections with other serotypes in the same individual [1].

Recently, the Asia Pacific region has been burdened with an increased threat of dengue. Thailand is experiencing the largest dengue epidemic in more than 20 years [7]. The escalating cases have also been observed in Bangladesh [8]. In the Philippines [9], Laos [10], and Myanmar [11], similar patterns of increased cases, severity, and mortality were observed. A study in Sri Lanka identified that the increasing number of dengue cases and the changes in the virulence of the virus were due to the shifting of genotypes and subtypes of the circulating DENVs [12]. This diversity amongst genotypes and serotypes is one of the major challenges in the development of tetravalent vaccines [13].

Dengue is endemic in Malaysia, and the first case was detected back in 1902 [6]. It is prevalent throughout the year although peak transmission occurs in the late monsoon season. Malaysia recorded the highest number of dengue cases through 2014 and 2016 [6], where 2015 observed a peak with 120,836 cases and 336 deaths, which might translate into one death daily [6]. Although the cause of this increase is not known, this might be due to improved notification rates as well as access to better diagnostic tools. However, the possible reasons for the increase in mortality remain obscure leading us to wonder whether the variation in serotype is the contributing factor to the increasing cases and mortality [14].

A similar pattern of increasing trend of dengue infection is also observed in Sabah which needs immediate measures to assess this regional issue. However, data on the serotype distribution in Sabah is still lacking [15]. This requires a comprehensive study on the epidemiology and circulating serotypes in Sabah to determine whether they are associated with increased virulence or whether other associated factors are responsible. The aim of this study is to determine the burden of dengue as well as to identify the circulating serotypes of DENV in Sabah during the past 6 years along with the temporal patterns and other factors that are associated with severe dengue. The information obtained from this study will be useful to formulate a policy for dengue control in this state.

## Methods

### Study area

The study was performed in Sabah, Malaysia, situated in the northern part of Borneo island. The size of the state is 73,904 km^2^ with a population of 3.9 million [16]. Sabah has land borders with the Malaysian state of Sarawak to the southwest and Indonesia’s Kalimantan region to the south. It also shares maritime borders with Vietnam to the west and the Philippines to the north and east. Sabah has abundant natural resources, and its economy is strongly export oriented. Its primary exports include oil, gas, timber, and palm oil. The other major industries are agriculture and ecotourism. The capital of Sabah is Kota Kinabalu, and the state is divided into Kudat, Interior, Sandakan, Tawau, and west coast divisions with a total of 23 districts.

### Case definitions

This paper followed WHO’s 2009 criteria for case definition of dengue fever and severe dengue [17]. Dengue is defined as a combination of two or more clinical findings in a person with fever. The clinical findings include nausea, vomiting, rash, aches and pains, a positive tourniquet test, leukopenia, and the following warning signs: abdominal pain or tenderness, persistent vomiting, clinical fluid accumulation, mucosal bleeding, lethargy, restlessness, and liver enlargement. The presence of a warning sign may predict severe dengue in a patient.

Severe dengue is defined as dengue with symptoms of either severe plasma leakage leading to shock or fluid accumulation with respiratory distress, severe bleeding or severe organ impairment such as elevated transaminases ≥ 1000 IU/L, impaired consciousness, or heart impairment.

### Data preparation

**Fig. 1:**
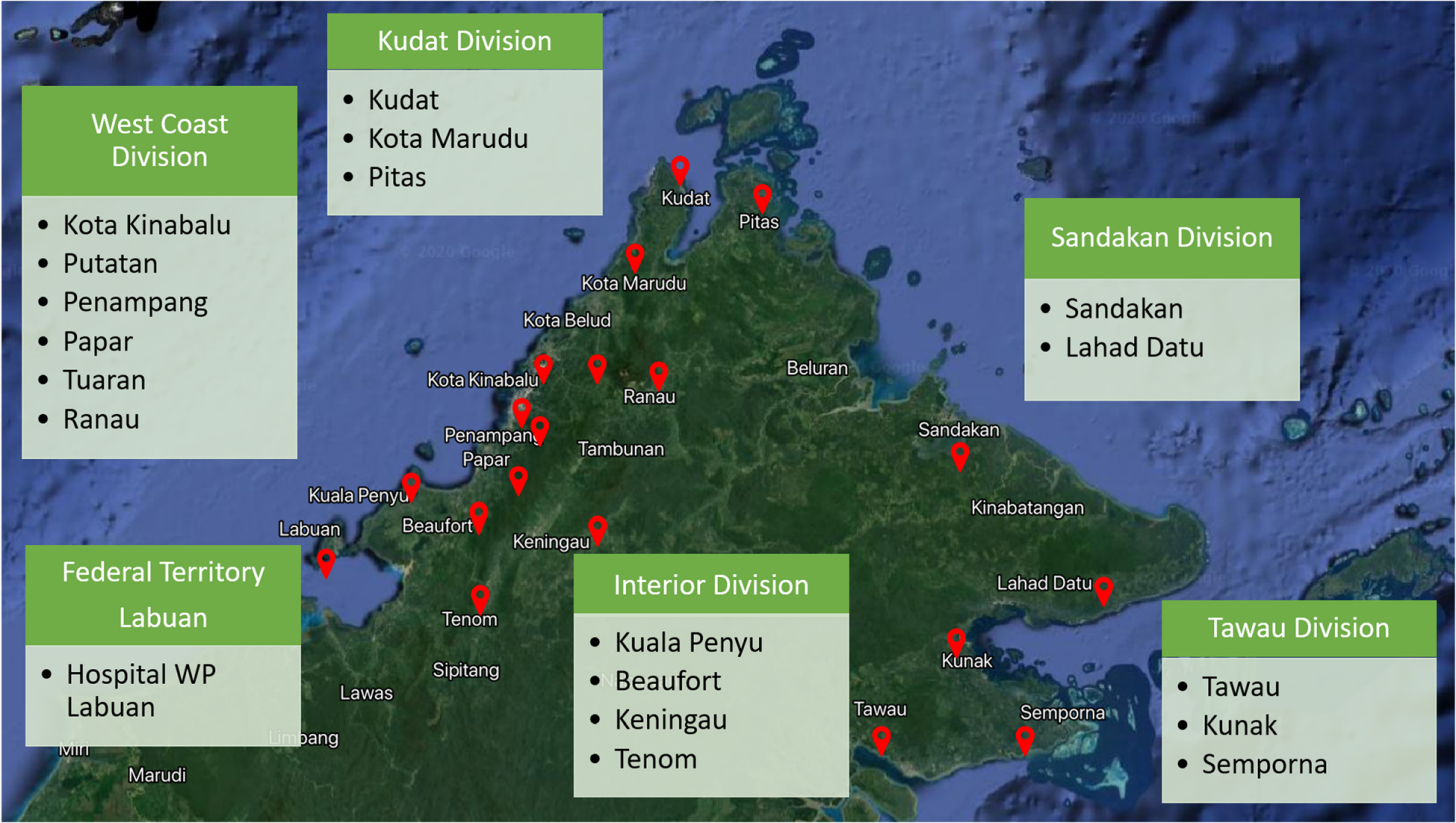
Map of Sabah showing the sentinel sites in different districts

Data on dengue cases and death from 2013 through 2018 were extracted from the database of dengue infection from Sabah State Health Department. Dengue NS-1 positive serum samples were sent to Kota Kinabalu Public Health Laboratory from various sites throughout Sabah for serotype determination under the virus serotype surveillance program as well as for diagnostic purposes. Dengue NS-1 assay was performed according to the manufacturer’s instructions using serum specimens. The rapid diagnostic test had a control line and a test line. The appearance of the test and control lines after a specified migration time (15–30 min) indicated a positive result. The appearance of the control line alone indicated a negative result. The technicians carrying out the evaluation of the test articles were blind to the DENV infection status of the panel of serum samples. At present, samples were obtained from 20 hospitals and 17 health clinics throughout Sabah (Fig. 1). Although Labuan is a federal territory, i.e. separate from the state of Sabah, but because of its geographical proximity to Sabah, samples are sent to Kota Kinabalu Public Health Laboratory for testing. Analysis of severe dengue was conducted for the samples obtained in 2018 only because severe dengue was recorded as a separate variable in the database as of 2018. Descriptive statistics, as well as multiple logistic regression, were performed to analyze the data using IBM SPSS Statistics software package (International Business Machines Corporation, Armonk, NY, USA).

### Serotyping

Serotyping was done at Kota Kinabalu Public Health Laboratory. Total viral RNA was extracted from serum samples using QIAamp Viral RNA Mini Kit (Qiagen Company, Hilden, Germany) according to the manufacturer’s instructions. All the serotypes of DENV were determined by real-time PCR using *ab*TES Den 4 (AITbiotech, Singapore) according to the manufacturer’s instructions using exacted RNA.

## Results

### Dengue burden

#### Prevalence of dengue in Sabah

**Fig. 2:**
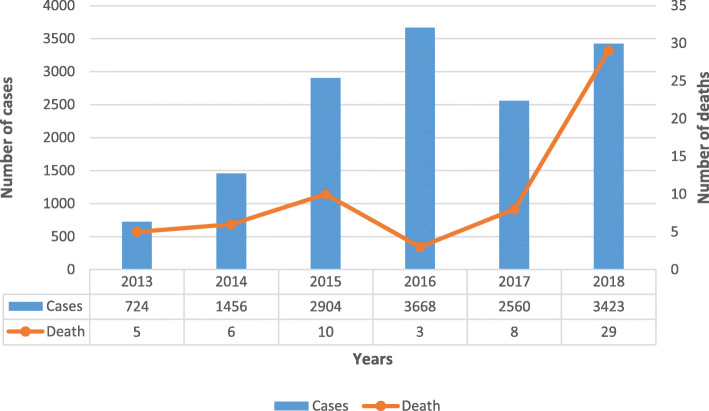
Yearly distribution of dengue cases and deaths from dengue in Sabah from 2013 through 2018. The number of dengue cases and the number of deaths are shown via a table format below the graph. Numbers on the left and right sides of the graph indicate the number of cases and deaths, respectively

In 2013, there were 724 dengue cases; however, from 2014, dengue cases increased exponentially to 1456 cases, which is almost double the number of cases from the previous year (Fig. 2). In 2015, the cases doubled even further to 2904. In 2016, the number of cases peaked at 3668 cases, and there was a brief decrease in cases to 2560 in 2017. These numbers did not stay low but increased to 3423 cases in 2018.

Unfortunately, increasing dengue cases have also led to increased dengue mortality (Fig. 2). The number of dengue deaths reported was 5 and 6 in 2013 and 2014, respectively. In 2015, the number of deaths increased to 10, but in 2016, it reduced to 3 deaths. This reduction did not last long as the numbers increased to 8 in 2017. The most shocking figure was reported in 2018 with a staggering total of 29 deaths reported. This shows an increase of 362% in 1 year (from 2017 to 2018).

#### Dengue deaths based on each district

The deaths based on districts are described in Table 1. From 2013 to 2017, the total death in Sabah ranged from 3 to 10; however, in 2018, it was 29. During these 6 years, the total death in Kota Kinabalu was 7; however, the total deaths in each district in the west coast was between 0 and 2. In the east coast districts, the total number of deaths was highest in Tawau with 10 deaths, followed by Semporna with another 10 deaths, and subsequently Lahad Datu with 8 deaths, followed by Sandakan which also reported 8 deaths.

**Table 1:** Number of dengue deaths in each district in Sabah from 2013 through 2018

District	2013	2014	2015	2016	2017	2018	Total
Kota Kinabalu	1	2	1	0	1	2	7
Penampang	0	0	1	0	1	0	2
Papar	0	0	0	0	0	1	1
Lahad Datu	0	1	0	1	1	5	8
Tawau	2	1	1	0	1	5	10
Beaufort	0	0	0	0	0	0	0
Semporna	0	0	3	0	2	5	10
Sandakan	1	0	3	1	0	3	8
Kudat	0	0	0	0	0	2	2
Pitas	0	0	0	0	0	0	0
Beluran	0	1	0	0	0	0	1
Kunak	0	0	0	0	1	5	6
Kota Marudu	1	0	0	0	0	0	1
Kinabatangan	0	0	0	0	0	0	0
Sipitang	0	0	0	0	0	0	0
Putatan	0	1	1	0	0	0	2
Ranau	0	0	0	1	0	0	1
Tuaran	0	0	0	0	1	1	2
Total	5	6	10	3	8	29	61

#### Dengue cases from multiple sites

A total of 3224 samples were obtained from all the multiple sites in Sabah (Table 2). In 2013, 2014, 2015, 2016, 2017, and 2018, a total of 182, 198, 379, 150, 964, and 1351 samples were collected, respectively. Age distribution of dengue was available for 2018 only. The majority of dengue cases were adults (603/1351, 44.6%), followed by adolescents (405/1351, 30.0%), and children (343/1351, 25.4%). According to Najri et al. [15], for age categories, adults were defined as those 20 years and above, adolescents were 10–19 years old, and children were under 19 years old.

**Table 2:** Number of samples collected from dengue patients from multiple sites in Sabah from 2013 through 2018

Division	District	Sentinel hospitals and clinics	2013	2014	2015	2016	2017	2018	Total
West coast division	Kota Kinabalu	Queen Elizabeth Hospital	139	129	209	30	440	307	1250
		Queen Elizabeth Hospital 2				1	3	11	15
		Sabah Women and Children Hospital		2	1	12	26	135	176
		Klinik Kesihatan Ibu Anak Jalan Kebajikan					1		1
		Klinik Kesihatan Likas					1		1
		Klinik Kesihatan Luyang	41	41	5	1	76	67	231
		Klink Kesihatan Menggatal		1					1
		Klinik Kesihatan Telipok			4	5	1		10
	Putatan	Klinik Kesihatan Putatan		7	63	8		8	86
	Penampnag	Klink Kesihantan Penampang			1				1
	Papar	Hospital Papar						3	3
	Tuaran	Hospital Tuaran					122	49	171
		Klinik Kesihatan Tamparuli		3	3	1	6	1	14
		Klinik Kesihatan Kiulu			1				1
	Ranau	Hospital Ranau			2		1	1	4
Kudat division	Kudat	Hospital Kudat		1	4	4	54	28	91
	Kota Marudu	Hospital Kota Merudu		2				1	3
		Klinik Kesihatan Tandek			1				1
	Pitas	Hospital Pitas	2	5				2	9
Interior division	Kuala Penyu	Hospital Kuala Penyu			2				2
	Beaufort	Hospital Beaufort		3	2	2			7
	Keningau	Hospital Keningau			1		3	2	6
		Klinik Kesihatan Bingkor						2	2
	Tenom	Hospital Tenom					1		1
Sandakan division	Sandakan	Hospital Duchess of Kent			67	73	18	12	170
		Klinik Kesihatn Sandakan			4				4
	Lahad Datu	Hospital Lahad Datu		3			27	121	151
		Klinik Kesihatan Lahad Datu			3			10	13
		Klinik Kesihatan Tungku						12	12
		Klinik Kesihatan Felda Sahabat						1	1
Tawau division	Tawau	Hospital Tawau		1	5	1	149	384	540
		Klinik Kesihatan Tawau						24	24
	Kunak	Hospital Kunak						56	56
	Semporna	Hospital Semporna			1	12	2	84	99
Federal territory Labuan		Hospital WP Labuan					33	30	63
Total each year			182	198	379	150	964	1351	3224

Among the sites, the highest number of samples was from the Queen Elizabeth Hospital situated in the Kota Kinabalu district as it is the largest tertiary care state hospital and has the largest population coverage in Sabah.

#### Severe cases in multiple sites

No severe case was detected in 2013 (Table 3). In 2014, three severe cases were detected out of the 198 cases in the health centers which is 1.5%. During 2015, 2016, and 2017, 2.1% (8/379), 2% (3/150), and 0.5% (5/964) of the total cases, respectively, were severe. In 2018, 10.8% (146/1351) of the cases were severe. During the whole study period, district wise, most of the severe cases were found in Tawau (110), followed by Lahad Datu (17), Kota Kinabalu (17), Sandakan (11), Semporna (9), and Labuan (1).

**Table 3:** Distribution of severe dengue cases in different districts of Sabah

Year	Number of severe cases	Number of case and name of district	Total
2013	0	–	182
2014	3	1-Lahad Datu 1-Tawau 1-Luyang (Kota Kinabalu)	198
2015	8	1-Tawau 3-Semporna 4-Sandakan	379
2016	3	1-Semporna 2-Sandakan	150
2017	5	2-Tawau 1-Labuan 1-Women and Children’s Hospital (Kota Kinabalu) 1-Queen Elizabeth Hospital (Kota Kinabalu)	964
2018	146	106-Tawau 16-Lahad Datu 5-Semporna 5-Sandakan 7-Women and Children’s Hospital (Kota Kinabalu) 7-Queen Elizabeth Hospital (Kota Kinabalu)	1351

### Circulating serotype in Sabah

**Fig. 3:**
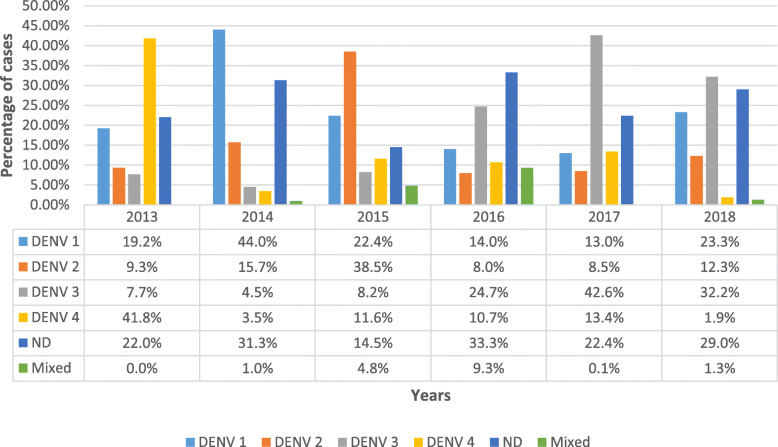
Predominant dengue virus serotype in Sabah during 2013–2018. DENV-1, DENV-2, DENV-3, and DENV-4 indicate the four serotypes of the dengue virus. ND indicates that the serotype could not be detected. Mix indicates two or more serotypes in the sample

In Sabah, the predominant circulating serotype in 2013 was DENV-4. In 2014, DENV-1 was predominant. Interestingly, in 2015, DENV-2 was dominant. Then, from 2016 up to 2018, DENV-3 became dominant (Fig. 3). Mixed serotypes ranged from 0 to 1% and abruptly increased to 5% in 2015. It then increased further to 9.3% in 2016, and from 2017 came back down to 0.1%. Undetermined serotype was between 14.5 and 33.3%. In 2018, a total of 17 samples were designated as containing mixed serotypes, two different serotypes present concurrently in one sample. Eight samples contained the combination of DENV 2 and DENV 3, seven samples contained the combination DENV 1 and DENV 3, and two samples contained the combination of DENV 1 and DENV 2.

### Monthly distribution of dengue serotype

**Fig. 4:**
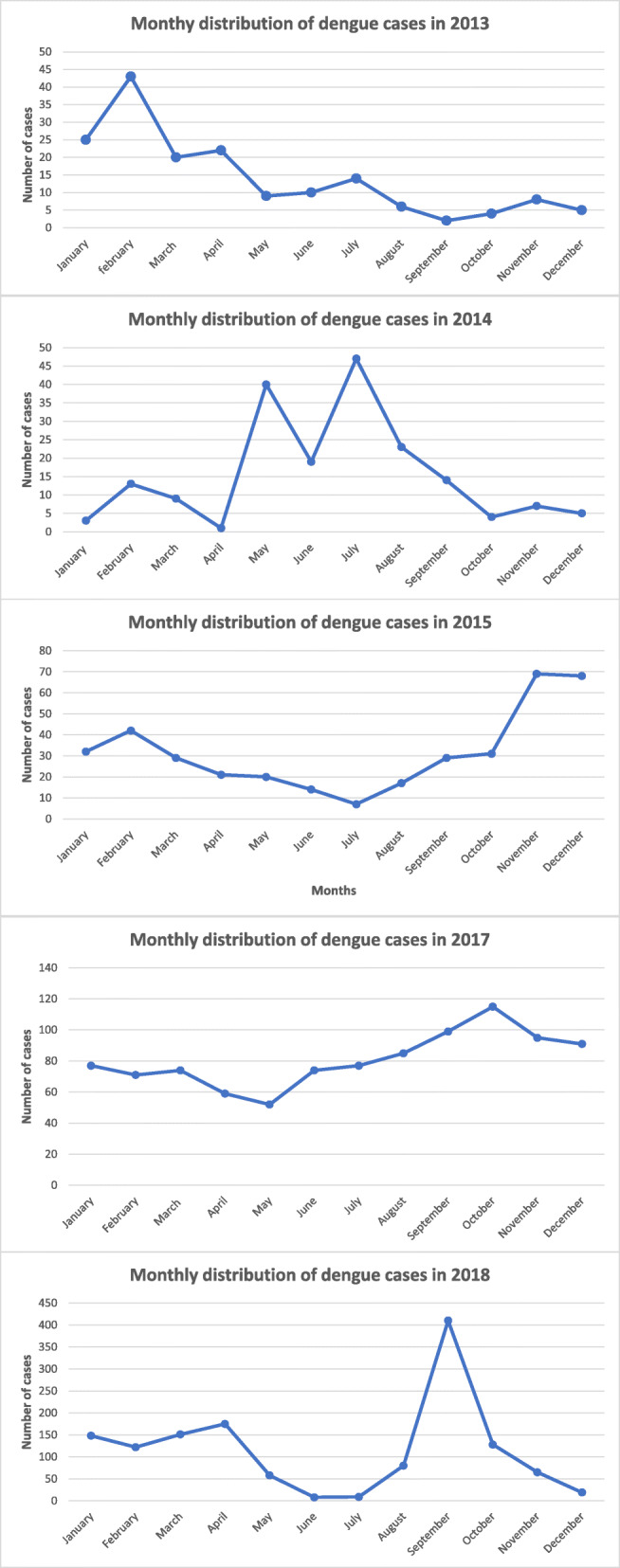
Monthly distribution of positive dengue virus cases in 2013, 2014, 2015, 2017, and 2018

In 2013, one peak was evident in February, whereas in 2014, two peaks were detected in May and July (Fig. 4). In 2015, again two peaks were noted, one in February and the other in November. In 2016, dengue data were not fully available and not included in this study. In 2017, the peak was in October. In 2018, again two peaks were noted in April and September.

### Associating factors for severe dengue

Our logistic regression analysis revealed that children were 6.5 times (95% CI 3.45–12.27, *p* = 0.00) more likely to develop severe dengue when compared to adults (Table 4). Patients who were infected with the mixed DENV serotypes were 13 times more likely to develop severe dengue (95% CI 3.67–46.01, *p* = 0.00), patients infected with the DENV-1 serotype were 1.8 times more likely to develop severe dengue (95% CI 1.21–2.88, *p* = 0.00), compared with those infected with DENV-2, 3, and 4 infections. It was also identified that cases reported from the eastern part of Sabah (Sandakan and Tawau divisions) were 5.2 times more likely to develop severe dengue (95% CI 2.74–9.79, *p* = 0.00) compared with cases from other parts of Sabah.

**Table 4:** The regression coefficient was determined to find the effects of a predictor variable on the severity of dengue cases. Since the *P* values are all < 0.05, therefore, the variables are significant to the model. Wald statistics identified that each factor is significant and contributes to the prediction model

Variable	Regression coefficient	Adjusted odds ratio (95% confidence interval)	Wald statistics	*P* value
Age (0–9 years)	1.87	6.5 (3.45–12.27)	33.36	< 0.001
Mixed dengue virus serotype	2.56	13 (3.67–46.01)	15.78	< 0.001
Dengue virus serotype-1	0.62	1.8 (1.21–2.88)	7.88	0.005
Districts of east coast	1.65	5.2 (2.74–9.79)	25.64	< 0.001

## Discussion

Although Sabah dengue cases are lower compared to other states in Peninsular Malaysia [18], this situation is changing, and an increasing trend of dengue infection has been observed. The highest number of dengue cases was reported at Queen Elizabeth Hospital, Kota Kinabalu. This can be attributed to the fact that it is the largest tertiary care state hospital and has the largest population.

However, regardless of that fact, an increasing trend of dengue can be observed in other hospitals as well, for example, from Hospital Tawau, Hospital Lahad Datu, and Hospital Semporna. These results further solidify the fact that dengue cases in Sabah are indeed increasing. The number of cases from Sabah Women and Children’s Hospital is also increasing yearly. This is a tertiary care hospital mainly for children and situated in Kota Kinabalu. This increasing number is worrying as it indicates more children are suffering from dengue. A study conducted showed that 67% of the children in Southeast Asia are vulnerable to dengue [19]. It supports our results that children suffer more from severe dengue than other age groups.

It has also been identified that the lifetime risk of getting dengue is more than 90% in Malaysia [19]. Those infected require immediate medical consultation. This can pose as a serious economic burden as the household cost of a single dengue case in Malaysia is about US$ 365 (MYR 1460) [20]. At the country level, the economic cost is more. It was estimated that about US$ 73.5 million (MYR 294 million) is spent yearly on dengue vector control only [21]. This amount constitutes 0.03% of the country’s GDP and 1.2% of the total government funding for health care in Malaysia (MYR 24 billion) [21]. If the trend of dengue increases, then it will increase the cost proportionately.

The results obtained from this study showed that dengue is prevalent in Sabah throughout the year; however, the peak varies from year to year. The climate in Sabah is considered equatorial; however, the climatic conditions are different in areas such as the highlands when compared to coastal areas. Although the hottest time of the year is between May and September, the temperature difference between this time and the rest of the year is very minimal. Only two seasons are distinguishable, which are rainy and dry. Although rain is a possibility year-round, the rainy season in most of Sabah is from October to February. The rest of the year is considered the dry season. However, the west coast of Sabah starts to have a higher rainfall from May until the end of the year, while the east coast is relatively stable during June and July with afternoon showers. Therefore, more study is needed to determine whether the seasonal variation of dengue differs from place to place.

Seasonal appearance of mosquitoes is one of the important factors for dengue transmission. A study in Singapore showed that infected *Aedes aegypti* appeared much earlier, compared to *Aedes albopictus*, approximately as early as 6 weeks before the occurrence of an outbreak [22]. Furthermore, all outbreaks reported from Taiwan for two decades showed that *A. aegypti* was more critical to the transmission of dengue viruses than was *A. albopictus* [23]. Perhaps, dengue outbreaks can only occur when *A. aegypti* exists or it is the predominant species [24].

But generally speaking, it can be deduced from the data that the peak of dengue infection is in the late monsoon seasons, which is throughout October to February in Sabah. This period is also coinciding with the national school holidays during which time families travel to different parts of Malaysia to meet relatives or for leisure. This might help in further spreading dengue to different places as there is an association of human movements with an increase in dengue transmission [25].

All four dengue serotypes have been reported from Malaysia [26]. National-level data, i.e. cumulative data from the whole of Malaysia, showed that in 2013, DENV2 was predominant, followed by a shift to DENV-1 after the second half of 2014 [27]. While in Sabah, the predominant serotypes in 2013 and 2014 were DENV-4 and DENV-1, respectively. Again, at the national level, DENV-1 continued to be predominant in 2015 and 2016, and DENV 2 in 2017 [6]. Interestingly, in 2015, DENV-2 was predominant in Sabah, and from 2016 up to 2018, DENV-3 became predominant.

Therefore, the predominant serotypes circulating in Sabah are different from the national level serotypes. This may be attributed to the fact that Sabah is approximately 1600 km from peninsular Malaysia and close to Sarawak and countries such as Indonesia, the Philippines, and Brunei. Although there were no published reports on the serotype distributions in Brunei and Sarawak, in Indonesia, the predominant serotype was DENV-3 in 2015 [28]. Similarly in Kalimantan, which is the closest Indonesian province to Sabah, DENV-3 was reported as the predominant DENV in 2015 to 2016 [29], which resembled Sabah’s serotype dominance at the time. In the Philippines, the predominant serotype in 2015 was DENV-2 [30], which again was similar to Sabah.

The number of severe cases increased considerably in 2018 which was a 96% increase from the previous year. It was also observed that many severe cases were from the east coast of Sabah, mainly Tawau, Lahad Datu, Semporna, and Sandakan. However, most dengue cases reported were from the western coast, mainly the district of Kota Kinabalu. Based on the results obtained from this study, we have identified that children infected with mixed serotypes and DENV-1 serotypes were significantly associated with severe dengue as compared to those infected with DENV-2, 3, and 4. Some studies have shown that there is a strong correlation between DENV-1 with severe outcomes when compared with DENV-2 and DENV-3 [6]. In this study, we identified that cases from the eastern coast of Sabah were significantly associated with severe dengue when compared to other parts of the state. A previous study also identified that the east coast of Sabah bears the brunt when it comes to severe dengue cases [18] similar to the findings of this study. This shows that these three factors can be incorporated into a predictive model for severe dengue. The case fatality rate (CFR) of dengue in Malaysia varied from 0.18 to 0.28% during 2013–2018 (http://idengue.arsm.gov.my/. Accessed 6 July 2020). For Sabah, the CFR in 2013 was 0.69%; in 2014, it was decreased to 0.41%; and in 2015 and 2016, it further decreased to 0.344% and 0.08%, respectively. However, in 2017, the CFR increased to 0.31%, and in 2018, it was 0.85%. Although the CFR of Sabah was higher than the national level, these levels were below 1% as targeted by the WHO.

Possibly, dengue has been around in Sabah for a long time as the vector has been known to be present in Sabah from as early as 1920 [31]. However, the increasing number of dengue cases has only started lately, and the reasons behind it are largely unknown. Dengue is known as an urban disease as it strives in urban surroundings [32], and since rapidly expanding urban populations [33] can be observed in Sabah, it is thought that urbanization might be a factor for an increased incidence of dengue. Sabah is rapidly gaining economic progress with the state GDP increasing from 2.7% in 2011 to 8.2% in 2017 [34] and experiencing rapid urbanization in the form of large physical growth of urban areas along with environmental changes leading to economic development [35]. The dynamic movement of people and the change in land use are mediators for human-mosquito interactions, which indirectly expands mosquito habitats [36]. These figures interestingly reflect the trend of increasing dengue cases from 2013 to 2018 in Sabah, indicating perhaps a correlation between both trends.

## Conclusions

Dengue burden is high in Sabah, with increasing mortality especially in children, infection with mixed serotypes, and cases from the east coast being the biggest contributors to severe dengue. More extensive research needs to be conducted to identify the genomic makeup of these virulent serotypes and to identify how it contributes to the severity of dengue. Understanding this might open a new avenue of supportive care which can be given earlier to reduce the number of deaths from dengue. The phenomenon of the mixed serotype must be studied more extensively along with DENV-1. With added information, appropriate changes can be made, and better strategies can be adopted to reduce the mortality rate especially in the early detection of potentially severe dengue cases. These factors can hopefully be incorporated into a severe dengue predictive model to ensure early detection.

This supports the hypothesis that the virus strain and small genotypic changes may be important modifying factors for severity, and hence, phylogenetic analysis is important when interpreting serotype-specific data [14]. The early detection of circulating serotypes could be an important approach to prevent severe clinical outcomes during dengue outbreaks [15]. This is important not only for dengue-endemic countries but also from the clinical aspect in non-endemic countries. This can aid in the management of travelers infected with various dengue viruses circulating globally [14].

## Data Availability

The datasets used and/or analyzed during the current study are available from JJFD, Kota Kinabalu Public Health Laboratory, on reasonable request.

## Abbreviations

NS-1: Non-structural protein 1
DENV: Dengue virus
PCR: Polymerase chain reaction
RNA: Ribonucleic acid
CI: Confidence interval
OR: Odds ratio
MYR: Malaysian Ringgit
US$: United States of America dollar
GDP: Gross domestic products
WHO: World Health Organization
RNA: Ribonucleic acid
PCR: Polymerase chain reaction
